# Expression of chemokine receptors on peripheral blood lymphocytes in multiple sclerosis and neuromyelitis optica

**DOI:** 10.1186/1471-2377-10-113

**Published:** 2010-11-11

**Authors:** Akiyuki Uzawa, Masahiro Mori, Sei Hayakawa, Saeko Masuda, Fumio Nomura, Satoshi Kuwabara

**Affiliations:** 1 Department of Neurology Graduate School of Medicine, Chiba University, Chiba, Japan; 2 Department of Molecular Diagnosis, Graduate School of Medicine, Chiba University, Chiba, Japan

## Abstract

**Background:**

The role of different chemokine receptors in the pathogenesis of multiple sclerosis (MS) has been extensively investigated; however, little is known about the difference in the role of chemokine receptors between the pathogenesis of neuromyelitis optica (NMO) and MS. Therefore, we examined the expression of chemokine receptors on peripheral blood lymphocytes (PBL) in MS and NMO.

**Methods:**

We used flow cytometry to analyse lymphocyte subsets in 12 patients with relapsing NMO, 24 with relapsing-remitting MS during relapse, 3 with NMO and 5 with MS during remission.

**Results:**

Compared with healthy controls (HC), the percentage of lymphocytes in white blood cells was significantly lower in NMO and MS patients. The percentage of T cells expressing CD4^+^CD25^+ ^and CD4^+^CD45RO^+ ^was higher, while that of CD4^+^CC chemokine receptor (CCR)3^+ ^(T helper 2, Th2) was significantly lower in MS patients than in HC. The ratios of CD4^+^CXC chemokine receptors (CXCR)3^+^/CD4^+^CCR3^+ ^(Th1/Th2) and CD8^+^CXCR3^+^/CD8^+^CCR4^+ ^(T cytotoxic 1, Tc1/Tc2) were higher in MS patients than in HC. The percentage of CD8^+^CXCR3^+ ^T cell (Tc1) and CD4^+^CXCR3^+ ^T cell (Th1) decreased significantly during remission in MS patients (*P <*0.05). No significant differences were identified in the expression of the chemokine receptors on PBL in NMO patients compared with MS patients and HC.

**Conclusions:**

Th1 dominance of chemokine receptors on blood T cells and the correlation between CXCR3^+ ^T cell (Th1 and Tc1) and disease activity in MS patients were confirmed by analysing chemokines receptors on PBL. In contrast, deviation in the Th1/Th2 balance was not observed in NMO patients.

## Background

Chemokines attract various types of leukocytes to sites of infection and inflammation and act as immunoregulatory molecules in driving T-helper (Th)1/Th2 responses [1]. The role of different chemokines and chemokine receptors in the pathogenesis of multiple sclerosis (MS) has been extensively investigated [2-5]; however, little is known about the role of chemokine receptors in the pathogenesis of neuromyelitis optica (NMO).

NMO is an immune-mediated inflammatory disorder of the central nervous system and predominantly affects the optic nerves and spinal cord [6]. Recent evidences such as the discovery of serum anti-aquaporin-4 (AQP4) antibody in NMO patients [7] (now recognised as a specific biomarker for NMO), differences in pathology [8], neuroimaging [9], cytokine profiles [10,11] and responses to some immunotherapies between MS and NMO [12], supports the hypothesis that NMO is distinct from MS.

In the present study, we report the expression of chemokine receptors on peripheral blood lymphocytes (PBL) in NMO and MS during relapse compared with healthy controls (HC) to determine differences in the expression of chemokine receptors between these disorders.

## Methods

### Patients

This study included 12 Japanese patients with relapsing NMO fulfilling Wingerchuk's revised criteria [6] that uses seropositivity of the anti-AQP4 antibody [13] instead of NMO-IgG. In addition, the study included 24 patients with relapsing-remitting MS fulfilling McDonald's criteria [14]. Their whole blood samples were obtained during relapse, which was defined as the period within one month of clinical relapse and before beginning relapse treatment. Blood samples from 25 healthy participants (9 women and 16 men; mean age, 32.7 years) served as controls. In addition, blood samples during remission were obtained from only 3 of the 12 NMO patients and 5 of the 24 MS patients.

We also reviewed the clinical characteristics of NMO and MS patients such as gender, age, disease duration, expanded disability status scale (EDSS) and the proportion of patients receiving immunomodulating treatment.

Ethics approval was granted by the Ethics Committee of Chiba University School of Medicine, Chiba, Japan. All study subjects gave informed consent for their participation.

### Flow cytometry

Blood samples collected in heparinised tubes were washed with phosphate buffered saline (PBS), supplemented with 0.5% foetal calf serum and resuspended in PBS with 0.5% foetal calf serum. Subsequently, the aliquots of the cell suspensions were double-stained with fluorescein isothiocyanate- and phycoerythrin-labelled monoclonal antibodies for 30 min at 4°C in the dark. Table 1 summarises the combinations of monoclonal antibodies used to identify chemokine receptor-positive cells as well as for other lymphocyte subsets. After lysing the red blood cells with FACS Lysing Solution (BD Biosciences, San Jose, CA, USA.), the cells were washed with PBS and resuspended in 0.5 ml PBS. IgG1 coupled to fluorescein isothiocyanate and IgG2a coupled to phycoerythrin were used as negative controls. The percentages of chemokine receptor-positive cells or lymphocyte subsets were obtained using a FACScan (BD Biosciences).

**Table 1:** Combinations of monoclonal antibodies used.

Chemokine receptor-positive cells	Other lymphocyte subsets
FITC-labelled PE-labelled	FITC-labelled PE-labelled
CCR3^c ^× CD4^b^	CD3^a ^× CD19^a^
CD4^b ^× CCR4^a^	CD3^a ^× CD4^a^
CCR5^c ^×CD4^b^	CD3^a ^× CD8^a^
CXCR3^c ^× CD4^b^	CD4^b ^× CD25^a^
CD8^b ^× CCR4^a^	CD45RA^a ^× CD4^b^
CXCR3^c ^× CD8^b^	CD4^b ^× CD45RO^a^

Monoclonal antibodies were purchased from ^a^BD Biosciences, San Jose, CA, USA, ^b^Beckman Coulter, Marseille, France, or ^C^Dako Japan, Kyoto, Japan,

FITC = fluorescein isothiocyanate; PE = phycoerythrin; CCR = CC chemokine receptor; CXCR = CXC chemokine receptor.

### Statistical analysis

For baseline variables, the groups were compared using Fisher's exact test for categorical outcomes, the Mann-Whitney *U *test for continuous variables and paired t-test for paired continuous measures. All comparisons were planned, tests were two-sided and *P *values < 0.05 were considered to be statistically significant. In addition, multiple testing problems were resolved by applying the Bonferroni correction on the computed *P *values to reduce type I errors. All statistical analyses were performed using SPSS 16.0J (SPSS Japan Inc., Tokyo, Japan).

## Results

### Clinical characteristics of NMO and MS patients

Clinical characteristics of 12 NMO and 24 MS patients are as described below. The proportion of female patients (NMO, men:women = 0:12; MS, men:women = 5:19; *P *= 0.11), age (NMO, 48.3 ± 13.8 years old [mean ± SD]; MS, 33.9 ± 10.0; *P *= 0.04) and EDSS (NMO, 8.0 [range 2.0-9.0]; MS, 3.0 [1.5-7.5]; *P *< 0.001) at the blood sampling were higher in NMO than in MS patients. The proportion of patients who received immunomodulating therapy was not statistically different between NMO and MS patients (33% in NMO, 1 patient with interferon beta and 3 with oral prednisolone; 21% in MS, 4 patients with interferon beta and 1 with oral prednisolone).

### Lymphocytes subsets in NMO and MS patients

**Figure 1:**
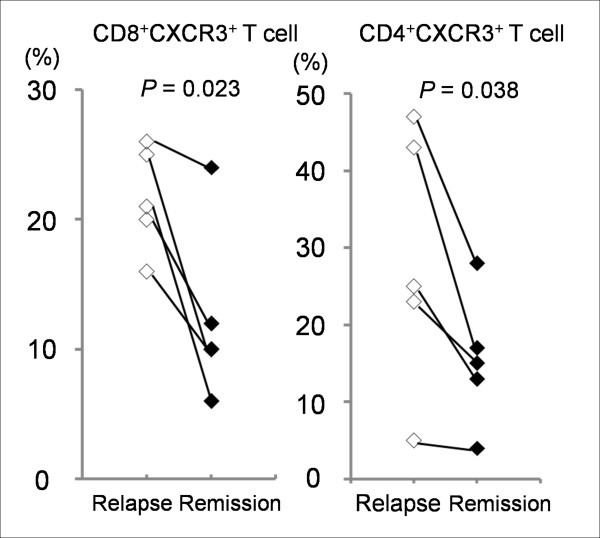
**Sequential data of CXCR3^+ ^T cells in MS patients**. The percentages of CD8^+^CXCR3^+ ^T cells (cytotoxic T type1 cells) and CD4^+^CXCR3^+ ^T cells (helper T type 1 cells) at the relapse and remission phase in 5 MS patients. Both percentages of CD8^+^CXCR3^+ ^and CD4^+^CXCR3^+ ^T cell decreased at remission phase in all 5 MS patients (*P *= 0.023 and 0.038).

White blood cell (WBC) counts were 7352 ± 732/μl (mean ± SE), 7492 ± 513 and 5864 ± 274 in MS patients, NMO patients and HC, respectively. WBC counts were significantly elevated in NMO and MS patients than in HC. Compared to HC, the percentage of lymphocytes was significantly lower in both NMO and MS patients. The percentage of T cells expressing CD4^+^CD25^+ ^and CD4^+^CD45RO^+ ^was significantly higher, while the percentage of T cells expressing CD4^+^CC chemokine receptor (CCR)3^+ ^(Th2 cells) was significantly lower in MS patients than in HC. The ratios of CD4^+ ^CXC chemokine receptors (CXCR)3^+^/CD4^+^CCR3^+ ^indicating Th1/Th2 and CD8^+^CXCR3^+^/CD8^+^CCR4^+ ^indicating cytotoxic T (Tc) 1/Tc2 was significantly higher only in MS patients. Although not statistically significant after applying the Bonferroni correction, the percentages of CD4^+^CXCR3^+ ^T cells (Th1 cells; *P *= 0.039), CD8^+^CXCR3^+ ^T cells (Tc1 cells; *P *= 0.020) and the ratio of CD4^+^CXCR3^+^/CD4^+^CCR4^+ ^indicating Th1/Th2 (*P *= 0.023) tended to be higher in MS patients compared with HC. CD8^+^CCR4^+ ^T cells (Tc2 cells; *P *= 0.017) and the ratio of CD4^+^CCR5^+^/CD4^+^CCR4^+ ^indicating Th1/Th2 (*P *= 0.041) tended to be lower in MS patients than in HC (Table 2). After applying the Bonferroni correction, NMO patients did not show any significant differences in the expression of the chemokine receptors on PBL compared with MS patients and HC. The analysis of patients between the relapse and remission phases revealed significantly decreased percentages of CD4^+^CXCR3^+ ^Th1 cells (*P *= 0.038) and CD8^+^CXCR3^+ ^Tc1 cells (*P *= 0.023) at the remission phase in all 5 MS patients (Figure 1). None of the lymphocyte subsets were correlated with clinical parameters such as EDSS, disease duration or anti-AQP4 antibody titre.

**Table 2:** Lymphocyte subsets (%) on blood of patients with NMO and MS

PB measurements (%)	Lymphocyte subsets	NMO (n = 12)	MS (n = 24)	NC (n = 25)	*P *value		
					
					NMO vs NC	MS vs NC	NMO vs MS
Lymphocyte		18.8 ± 1.8	25.7 ± 2.0	35.4 ± 1.8	<0.001*	0.001*	0.009*
CD3+	T cell	73.5 ± 2.6	70.2 ± 1.7	66.1 ± 1.7	0.039	0.071	0.486
CD3+CD4+	Th/Ti	40.8 ± 2.9	41.6 ± 2.2	38.8 ± 1.3	0.212	0.045	0.837
CD3+CD8+	Ts/Tc	31.5 ± 2.7	28.4 ± 1.7	26.4 ± 1.4	0.205	0.321	0.767
CD3+CD19+	B cell	15.4 ± 2.0	17.0 ± 1.2	17.3 ± 0.9	0.527	0.810	0.600
CD4+CD25+		16.8 ± 2.3	17.0 ± 1.4	11.4 ± 0.9	0.051	<0.001*	0.674
CD4+CD45RO+	Memory T	28.0 ± 2.4	27.1 ± 1.6	23.0 ± 0.9	0.039	0.007*	0.810
CD4+CD45RA+	Naïve T	22.9 ± 2.7	26.9 ± 2.0	25.8 ± 1.5	0.427	0.656	0.289
CD4+CXCR3+	Th1	23.9 ± 3.5	24.9 ± 2.4	19.6 ± 1.9	0.188	0.039	0.722
CD4+CCR5+	Th1	0.7 ± 0.2	1.0 ± 0.3	1.4 ± 0.3	0.112	0.062	0.730
CD4+CCR4+	Th2	25.2 ± 2.1	26.9 ± 1.9	26.4 ± 1.4	0.602	0.785	0.470
CD4+CCR3+	Th2	1.1 ± 0.2	0.9 ± 0.2	1.8 ± 0.2	0.022	0.001*	0.272
CD8+CXCR3+	Tc1	21.1 ± 4.1	18.3 ± 1.0	15.4 ± 1.9	0.183	0.020	0.844
CD8+CCR4+	Tc2	9.5 ± 2.4	6.9 ± 0.7	9.6 ± 0.9	0.355	0.017	0.952
CD4+CXCR3+/CD4+CCR4+	Th1/2	1.0 ± 0.1	1.0 ± 0.1	0.8 ± 0.1	0.122	0.023	0.908
CD4+CXCR3+/CD4+CCR3+	Th1/2	31.4 ± 9.4	23.1 ± 2.4	13.7 ± 1.9	0.035	0.007*	0.966
CD4+CCR5+/CD4+CCR4+	Th1/2	0.03 ± 0.01	0.03 ± 0.01	0.06 ± 0.01	0.093	0.041	0.646
CD4+CCR5+/CD4+CCR3+	Th1/2	0.7 ± 0.2	1.2 ± 0.3	0.8 ± 0.1	0.520	0.754	0.453
CD8+CXCR3+/CD8+CCR4+	Tc1/2	3.4 ± 0.6	3.4 ± 0.5	2.0 ± 0.3	0.056	<0.001*	0.738

Values indicate mean ± standard error, PB: peripheral blood, NMO: neuromyelitis optica, MS: multiple sclerosis, HC: healthy control, Th: helper Tcell, Ti: inducer T, Ts: suppressor T, Tc: cytotoxic T, CCR: CC chemokine receptor, CXCR: CXC chemokine receptor. *Statistically significantly even after multiple comparison adjustment.

## Discussion

In this study, we analysed the expression of chemokine receptors on circulating PBL during relapse in NMO and MS patients and compare it with that of HC. We confirmed the dominance of the Th1- and Tc1-related chemokine receptors in MS patients; however, no remarkable differences were observed between NMO and HC patients.

Th1 and Th2 subsets of lymphocytes can be characterised by their expression of chemokine receptors. CXCR3 and CCR5 are associated with a Th1 phenotype, whereas CCR3 and CCR4 are expressed preferentially on activated Th2 cells [15]. Several studies have reported Th1 dominance over the Th2 response associated with chemokine receptors in MS, increased percentages of T cells expressing CXCR3 and CCR5 chemokine receptors on PBL and cerebrospinal fluid (CSF) in the active phase of MS and the identification of CXCR3^+ ^and CCR5^+ ^T cells in active demyelinating MS brain lesions [2,3]. Th1 dominance over the Th2 response is also indicated by decreased expression of the chemokine receptor CCR4 on blood T cells and brain tissue [4,5]. The results of our study indicates higher expression of Th1-associated chemokine receptors CD4^+^CXCR3^+ ^on peripheral T cells and lower expression of Th2-associated chemokine receptors such as CD4^+^CCR3^+ ^in MS patients than in HC, which is in accordance with the results of the above-mentioned reports. Ratios of CD4^+^CXCR3^+^/CD4^+^CCR4^+ ^and CD4^+^CXCR3^+^/CD4^+^CCR3^+ ^were higher in MS patients than HC, which also indicates Th1 dominance in balancing Th1/Th2 responses. Furthermore, we confirmed higher percentages of peripheral CD4^+^CD25^+ ^(regulatory or activated T cells) and CD4^+^CD45RO^+^T cells (memory T cells) in MS patients than in HC, which is in accordance with previous studies [16,17].

Our results estimated that the ratios of CD8^+^CXCR3^+^/CD8^+^CCR4^+^, indicating Tc1/Tc2, were significantly higher in MS patients than in HC. In addition, the percentage of CD4^+^CXCR3^+ ^and CD8^+^CXCR3^+ ^T cells was correlated with disease activity in MS. The roles and functions of chemokine receptors on CD8^+ ^T cells remain to be elucidated. CD8^+^CXCR3^+ ^T cells are associated with migration and differentiation of memory T cells [18] and also have a similar function as regulatory T cells (Treg) [19]. Our results indicate Tc1 dominance over Tc2 in MS, which may reflect the abnormality of Treg in MS.

To the best of our knowledge, no studies have reported the expression of chemokine receptors on PBL in NMO patients. A few studies have investigated chemokine levels in NMO, in which CXCL10/IP-10 and CCL17/TARC levels were significantly elevated in NMO patients [20] and CXCL-8/IL-8, CCL4/MIP-1β and CCL2/MCP-1 levels showed significant elevation in opticospinal MS patients (part of them are considered NMO) compared with normal subjects [10].

Recent studies have shown that anti-AQP4 antibody and complements play a critical role of in the pathogenesis of NMO [21]. Thus, we expected the up-regulated expression of Th2-related chemokine receptors in NMO; however, no remarkable deviation in the Th1/Th2 balance was observed in this study. Recent studies have revealed that Th17-related cytokines and chemokines play a dominant role in the pathogenesis of NMO from elevated protein levels of IL-17 and IL-8 in the CSF of opticospinal MS patients [10] and IL-6 in the CSF of NMO patients [11]. Th17 cells belong to a distinct lineage of Th1 and Th2 cells, which are responsible for organ-specific autoimmune diseases. Although the association between Th17 cells and NMO remains unclear, Th17 related-responses may be more important than Th1-and Th2-related responses in NMO pathogenesis. Hence, no remarkable changes might be seen in the Th1/Th2-related chemokine receptors on peripheral T cells. However, our study has a limitation because the subject was chemokine receptors on only PBL, and not on CSF, and thus we were unable elucidate the complete Th1/Th2 balance in the pathogenesis of MS and NMO.

In this study, some patients received oral prednisolone treatment. It has been reported that high-dose intravenous methylprednisolone reduced CD4^+^CXCR3^+ ^Th1 cells [17] and CCR5^+ ^Th1 cells in MS patients [22]. Hence, it can be suggested that steroid treatment affected the expression of chemokine receptors in this study. However, only one (4%) patient with MS and three (25%) with NMO received oral prednisolone at sampling PBL, and no statistical difference was found between the percentage of patients treated with steroid in NMO and MS. We hypothesise that steroid treatment had a relatively small effect on the results. Meanwhile, higher WBC counts and lower lymphocyte percentages in NMO and MS patients were possibly affected by immunomodulating therapy.

## Conclusions

In conclusion, we have identified several differences, such as Th1 and Tc1 dominance and the association between CXCR3^+ ^(Th1 and Tc1) and disease activity in T cell subsets on PBL, in MS patients compared with HC, but these differences did not appear in NMO patients. We cannot determine characteristic lymphocyte subsets on PBL involved in the pathophysiology of NMO. Although further studies will be required to determine the immunological features of NMO, these differences in PBL could be based on immunological differences between NMO and MS.

## Abbreviations

MS: multiple sclerosis; NMO: neuromyelitis optica; HC: healthy control; AQP4: anti-aquaporin-4; EDSS: expanded disability status scale; PBL: peripheral blood lymphocyte; WBC: white blood cell; helper T: Th; cytotoxic T: Tc; Treg: regulatory T; CCR: CC chemokine receptor; CXCR: CXC chemokine receptor; CSF: cerebrospinal fluid;

## Competing interests

The authors declare that they have no competing interests.

## Authors' contributions

AU drafted the manuscript and carried out the statistical analysis. MM diagnosed MS and NMO patients, collected blood samples and provided other information of the patients. SH and FN participated in the design of the study. MM and SK critically reviewed the manuscript. All authors have read and approved the final manuscript.

## Pre-publication history

The pre-publication history for this paper can be accessed here:

http://www.biomedcentral.com/1471-2377/10/113/prepub
